# Equity in AgeTech for Ageing Well in Technology-Driven Places: The Role of Social Determinants in Designing AI-based Assistive Technologies

**DOI:** 10.1007/s11948-022-00397-y

**Published:** 2022-10-27

**Authors:** Giovanni Rubeis, Mei Lan Fang, Andrew Sixsmith

**Affiliations:** 1 Department General Health Studies, Division Biomedical and Public Health Ethics, Karl Landsteiner University of Health Sciences, Dr.-Karl-Dorrek-Straße 30, 3500 Krems, Austria; 2 School of Health Sciences, University of Dundee Nethergate, DD1 4HN Dundee Scotland, UK; 3 Department of Gerontology, Simon Fraser University Vancouver Harbour Centre, British Columbia, Room, 2800, V6B 5K3 Vancouver, Canada

## Abstract

AgeTech involves the use of emerging technologies to support the health, well-being and independent living of older adults. In this paper we focus on how AgeTech based on artificial intelligence (AI) may better support older adults to remain in their own living environment for longer, provide social connectedness, support wellbeing and mental health, and enable social participation. In order to assess and better understand the positive as well as negative outcomes of AI-based AgeTech, a critical analysis of ethical design, digital equity, and policy pathways is required. A crucial question is how AI-based AgeTech may drive practical, equitable, and inclusive multilevel solutions to support healthy, active ageing.

In our paper, we aim to show that a focus on equity is key for AI-based AgeTech if it is to realize its full potential. We propose that equity should not just be an extra benefit or minimum requirement, but the explicit aim of designing AI-based health tech. This means that social determinants that affect the use of or access to these technologies have to be addressed. We will explore how complexity management as a crucial element of AI-based AgeTech may potentially create and exacerbate social inequities by marginalising or ignoring social determinants. We identify bias, standardization, and access as main ethical issues in this context and subsequently, make recommendations as to how inequities that stem form AI-based AgeTech can be addressed.

## Introduction

AgeTech refers to the use of technologies and services to support aging. The new generation of AgeTech encompasses emerging and advanced technologies in areas such as artificial intelligence (AI), robotics, machine learning, e-health, and mobile technologies to support the health, independence and well-being of older people (Sixsmith, 2021). AgeTech has been explored in terms of supporting older people to remain at home for longer (Verloo et al., 2020), to provide social connectedness (Baez et al., 2019), support wellbeing (Astell et al., 2016) and mental health (Andrews et al., 2019) and connect the older person to their wider community (Fleming et al., 2018). However, the use of technology to provide support for older people to age well in place may also bring with it the potential to increase inequalities in access to health and health outcomes for vulnerable and marginalised people. This is especially the case when the needs and resources of individuals as well as groups are not fully acknowledged, when their characteristics are reduced to oversimplified or stereotypical narratives, and when barriers to equitable access exist. Thus, technology design and its functional requirements necessitate deeper insight into the range of diverse factors that may shape its context of use.

Research has indicated a crucial need to fully understand who and in what ways emerging and advanced technology interventions such as AI can have positive benefits for older people, or further exacerbate experiences of marginalisation (Sixsmith, 2006). A critical discussion of ethical design, digital equity, and policy pathways is required if we are to fully understand the positive and negative intended and unintended consequences of AI as an AgeTech solution to drive practical, equitable, and inclusive multilevel solutions to support healthy, active ageing. Regulations and the accompanying policies are without a doubt crucial instruments when it comes to avert harm from vulnerable groups, safeguard their civil rights, and avoid marginalisation. However, apart from the rather passive or reactive view that regulations and policies should act as a protective shield, a more active approach could be taken in regard of technology design and implementation. In order for AI-based AgeTech to realise its full potential, the focus on equity is key. Equity – as it pertains to the changing digital landscape – is defined in this paper as fairness and equality of access and use of AgeTech regardless of the myriad social characteristics which one holds, that combined, may create social disadvantages. As a mechanism to help to ensure equity by this definition, the threshold of having meaningfully addressed AgeTech can be achieved by (Fang, 2018; Fang et al., 2019): (1) assessing the intended and unintended positive and negative consequences of AgeTech; (2) considering who is most likely to benefit from AgeTech; and (3) responding to the challenges of those who experience the most significant barriers to access and use.

To help to ensure AI-based AgeTech to truly support an independent lifestyle and improve the quality of life of older adults, these technologies need to be designed in a way that enables agency as well as social participation. Therefore, equitable access has to be a key feature of AI-based AgeTech. The precondition for this is to acknowledge the diversity of older adults and to tailor technologies to their multifaceted needs and resources. Equity should thus be an explicit aim of designing AI-based AgeTech, and not just seen as an extra benefit or minimum requirement. This approach does not only aim at protecting older adults against the possible negative effects of AI-based AgeTech. It actively addresses the social determinants of health as defined by the WHO as non-medical factors that influence health outcomes by shaping the daily life and social situation of a person (WHO, 2010) (such as age, ethnicity, gender, socioeconomic status, and level of education) which may create experiences of marginalisation. We will explore how a particular facet of AI systems - complexity management - can potentially create and exacerbate social inequities, and subsequently, make recommendations as to how inequities that stem form AI-based AgeTech can be addressed. Although clinical as well as legal issues may also arise in this context, our specific focus is on the primarily ethical aspects connected to complexity management.

Each of the aformentioned social determinants can be a contributing factor for marginalisation, yet in reality, we seldom see these determinants as sole factors to shape inequity. More often, they are mutually dependent. For example, lower education and belonging to a social minority often correlate with lower socioeconomic status, which in turn, can lead to poorer health outcomes in old age. Fang et al., (2019) have identified this as a “wicked” problem, in view of three key principles of intersectionality (Hankivsky, 2014):

1) Single traits such as age, gender, or ethnicity are insufficient when it comes to understanding individual experiences – as reducing individuals to a single trait results in the oversimplification of their lived accounts.

2) Social determinants are not objective or fixed categories, but are seen as fluid and flexible social constructs that vary depending on the contexts of time and place and are shaped social processes, structures, and power relations.

3) Social justice and equity are not merely add-ons, but have to be considered as crucial aspects when it comes to policy-making.

## Complexity, Complexity Management and AI

A main ethical issue when it comes to AI-based AgeTech in the light of interconnected social determinants is complexity management. AI-technologies are built to detect patterns in large data sets and derive algorithms for predicting future events or controlling processes. In AgeTech, AI-technologies face the inherent complexity and unpredictability of everyday behaviors, situations, and contexts. For AI systems to operate within the current technological limitations, it is essential that complexity is managed, which often means reducing complex aspects to simple factors. Complexity management thus means to align the data with a given framework for processing it, which often means to standardise, decontextualise, and quantify data. This can become a problem when qualitative and context-rich data is reduced to manageable data formats, thus losing some of its crucial features. This is not merely a technical, but primarily a social and ethical issue, since it affects the way we are dealing with diversity in society and the specific needs and resources of individuals. Complexity in this context however does not only refer to different contexts of use, but also to the variety of user characteristics. Here lies the connection between complexity and vulnerability: the diversity of users regarding the aforementioned social determinants causes complexity, which in turn poses a problem that AI-based systems try to resolve by reducing complexity. Therefore, AI-based AgeTech aimed to support the personalisation of services may inadvertently do the exact opposite and thus posing a risk to vulnerable groups.

In order to analyse ethical issues connected to complexity management, we suggest examining the way social determinants are represented in the data that is used, how social determinants are taken into consideration when processing data, and how social determinants may shape the use of technologies that operationalise the data. Therefore, we identify three dimensions of ethical issues: The inappropriate representation of social determinants may cause flawed or stereotypical concepts od user characteristics or *bias*. The context-insensitive procession of data, i.e. an oversimplifying or reductionist approach, may lead to *standardisation* of user characteristics. Ignoring social determinants in technology use may negatively affect *access* to these AI-based AgeTech.

Complexity management thus has three crucial aspects which we explore in more detail in the following sections and, subsequently, recommend strategies for dealing with the issues at hand:

- **Bias-** Bias in relation to certain social characteristics may be introduced into AI systems at different stages in the development and innovation process, such as in the AI training data that is used or decisions made regarding the commercialisation and marketing of products and services.
- **Standardization**- the problematic aspect here is the underlying assumption that objective parameters can be defined and operationalised to identify and respond to ambiguous behaviors and situations.
- **Access-** Some AI-based technologies are designed in a way which makes them difficult to use by some individuals and groups.

## Complexity Management and Bias

Bias is one of the most fiercely debated issues in AI-based technologies (Challen et al., 2019; Mittelstadt & Floridi, 2016; Neven, 2015; Safdar et al., 2020; Wong, 2020). Bias can be a result of complexity management in two ways. Firstly, algorithm-based systems strongly rely on training data that has been used in their development. These systems learn by processing large amounts of this training data, detecting connections and patterns, and inferring general rules. The quality of the training data thus determines the quality of the algorithm-based system. The training data is usually taken from large cohort studies (Jones et al., 2018), in which certain social groups are often notoriously underrepresented. As a consequence, the bias within the training data is transferred into the system, leading to a biased algorithm. Thus, the needs and resources of various social groups are not considered by the system.

Secondly, AI-based systems and hardware devices are produced on a certain scale in order to be cost-efficient. In order to make the product suitable for a certain mass of users, complexity management regarding concepts of old age is necessary. Further, by way of complexity management, the risk of so-called *age scripts* arises, consequently, stereotypical concepts of what it means to be older aged, regarding needs and resources of older adults, are inscribed into the technology.

### Training Data

AI-systems learn by inferring algorithms from data sets (Jones et al., 2018). In order to operationalise this, the systems are fed with training data, mostly taken from large cohort studies. The more good quality training data a system is fed with, the more accurate its algorithms become. That also means that the quality of the algorithm, its accuracy and discriminatory power, depends largely on the quality of the training data, *and lack thereof* can lead to bias. A bias within the training data is often perpetuated in the system’s algorithm, as bias and complexity management often go hand in hand. This is largely due to the fact that prior to processing for analysis, the data is decontextualised, meaning as part of data preparation, the data becomes separated from its specific spatial, temporal, *or social determinants*. Racial bias is a prominent example for the connection between complexity management in the shape of decontextualization and bias. Obermeyer et al. (2019) have demonstrated this connection in a striking way upon analysing an algorithm used within the US health services. The task of this algorithm was to identify people with complex health needs. The algorithm assessed individual health costs and predicts future costs. It followed the basic assumption that individuals where more health costs have been invested in the past, have higher health needs and should therefore have easier access to health services. As a result of challenges that stem from socio-structural, and historical issues, when less health spending are applied in certain groups (i.e., African-American people), the algorithm assigns them a lower risk-score. This example demonstrates that the inherent technological need to reduce complexity in order to process data efficiently may lead to bias and thus conflict with the basic goal of providing enhanced person-centred health care. The result is a harmful feedback loop whereby health disparities manifest themselves in training data that do not represent social realities. Biased algorithms and predictive models are subsequently built based on these training data which can in turn aggravate the structural discrimination already inherent in the healthcare system (Walsh et al., 2020).

### Age Scripts

AgeTech relies on specific concepts of age, a narrative that defines characteristics of older adults as well as what age means. These age scripts are written into AgeTech and define the scope and purpose of the technology (Peine et al., 2015). Age scripts can be developed based on various sources such as societal views, design traditions, or individual interpretations (Peine & Neven, 2021). This implies a certain risk for stereotypical or poorly-informed views about older adults, their needs and resources, and their desired way of life becoming the basic design framework of AgeTech. Usually, such ageist stereotypes present older adults as ‘problem focused’ which assumes older people as a homogeneous group with associated traits such as frail, vulnerable, and in need of help (Ayalon & Tesch-Romer, 2018). The notion of age is fundamentally viewed as a problem to be solved by AgeTech (Rubeis, 2020). Although enabling agency is the intended goal, limitations arise when fundamental concepts and purposes of AgeTech are not determined with the end users (Neven, 2015; Peine et al., 2015). The result is the emergence of a sense of ‘benevolent paternalism’ that predefines the appropriate lifestyle of older adults without including them in the design process (Manzeschke et al., 2016). This is especially the case when digital equity was not considered as part of the intended goal.

Focusing on equity may help to diversify training data as well as the basic scripts that are written into the technologies. It is especially important to acknowledge that older adults do not constitute one homogenous group, but a diverse population who are shaped by a complex interplay of social determinants.

## Complexity Management and Standardisation

Standardisation means that certain parameters must be predefined for AI based systems to process. For example, a crucial parameter in some systems for fall detection is gait pattern (Piloto et al., 2018). These particular systems measure the typical gait pattern of a person, and whenever the gait pattern changes and/or there is a deviation from the standard, the system interprets this as a sign for an impending fall. The system may then trigger an alarm and inform caregivers. The issue here is not so much the definition of a standard as such since it is defined on the basis of a person’s individual health data. What is problematic, is the fact that specific parameters are defined as objective indicators of health and well-being. This suggests that primarily qualitative categories like well-being can be easily quantified by choosing the right parameters. This is an oversimplification that ignores the rich contexts of a person’s attitude towards well-being, health, and others, which forces them to adapt to pre-fixed definitions of well-being.

In the fall detection example and as seen in other examples, this may not seem like an issue since it is quite reasonable to define gait pattern as a parameter for assessing the fall risk. Another example is systems that recognise whether shutters are open or closed, *and if closed*, how long they remain closed during daytime. In this case, systems that draw conclusions based on the mental health status of a person because closed shutters throughout the day may imply a mood change, or even a depressive episode. It becomes clear that in this case, complexity management (closed shutters equals depression) can lead to a standardisation of behaviour.

Standards and standardised parameters play a key role in all data-driven technologies. The scope and characteristics of data that are deemed as useful or viable depend on the parameters for measuring them. In the context of digital health, the main parameters are digital biomarkers. Biomarkers are biomedical or behavior-related indicators used to measure, predict, or evaluate health-related outcomes (Guthrie et al., 2019; Sim, 2019). Raw data as detected by sensors are of no use for health care professionals without predefined biomarkers that allow for scaled and contextualised data. Digital biomarkers are therefore crucial for personalised interventions such as AgeTech.

In AgeTech, digital biomarkers may be used in sensor technologies, either in smart home sensors or smart wearables, in order to measure and predict changes in symptoms and behavior. Digital biomarkers have to be defined, meaning that a certain trait has to be singled out and deemed as a viable indicator. This may not be problematic in a biomedical context, e.g., determining blood pressure as biomarker for cardiovascular health. However, even in this context, blood pressure would first and foremost be seen as one factor amongst others with which it interacts and thus constitutes cardiac health. Regarding individual behavior, it is even more difficult to define clear-cut biomarkers. One example is a framework for recognising and regulating emotions in older adults presented by Castillo et al. (2014). The framework consists of sensors that measure physiological signals, facial expression, and voice in order to determine the emotional status of a person. A social robot reacts to these emotions and the color, lighting, and music in the room is adapted in order to enable positive emotions. In this setting, certain facial expressions for example are defined as indicators for a certain emotional state. A certain colour scheme or music is defined as a means of regulating emotions. The underlying assumption is that clear-cut parameters can be defined for distinguishing between positive and negative emotions as well as for regulating the negative ones. This blurs the line between support and conditioning, i.e. the regulation of a person’s conduct. Conditioning signifies an action directed at creating a specific behavior that is deemed as appropriate or desirable. For example, some stakeholders may have a personal interest in reducing costs of health care services. Thus, instead of modifying health care services to address the specific needs of individuals, methods of conditioning could be applied in order to shape the individual to fit the most cost-effective service. Taking the aforementioned example, the use of technology for regulating emotion may be understood as a way to achieve a certain behavioural standard which offers cost-effectiveness as opposed to providing person-centred care and tailored services. The same method could be applied to condition people to eat healthy or exercise in order for cost-saving purposes.

Conditioning has already been recognised as a risk in AgeTech (Hummel & Braun, 2020; Manzeschke et al., 2016; Mortenson et al., 2015; Petrakaki et al., 2018; Rubeis, 2020). What is seldom discussed is the connection between epistemological and ethical aspects in this context (Morley & Floridi, 2020). According to this view, the problem resides in the underlying assumption that objective parameters can be defined for appropriate or desirable emotions and behavior. There is a certain practical and ethical risk of creating parameters deliberatively designed in order to produce a specific behavior – a type of nudging (Thanler & Sunstein, 2008) that contradicts to the goal of person-centred care. An equity-focused approach could minimise the risks of standardization by taking the diversity of user preferences, needs, and resources into account, thus providing a more balanced and person-centered baseline for technology design.

## Complexity Management and Access

Another ethical quandary of complexity management is that it manifests itself by way of optimal AI design with limited consideration for issues relating to access i.e., lack of cultural appropriateness. “Culture” in this context may refer to certain codes, concepts, and behaviors that stem from the ethnic identity of older adults. It may, however, also be understood in terms of the life experience of certain generations. As such, the way in which individuals have been exposed to technology across their lifespan influences their attitudes and behaviour towards AgeTech in old age. Technology that is designed without consideration for cultural appropriateness can mean that they are more difficult to access resulting in reduced uptake.

Aligned with the issue of access is usability, and links to cultural appropriateness. For example, digital literacy, more precise the lack of it, can be an access barrier when it comes to AI-based AgeTech. This is especially the case with technologies that require direct interaction, like human-machine interfaces (HCI) or technologies for self-management and self-monitoring common in mHealth and telehealth (Fang et al., 2018). The lack of experience with digital technologies or affinity towards their use may thus prevent older adults from accessing them (Fang et al., 2019). Empirical evidence shows that digital literacy and health outcomes correlate with education, age, gender, and socioeconomic status (Ang et al., 2021).

The need for complexity management in AI-based AgeTech also affects access to AgeTech. One important factor in this regard is diversity within the older population (Fang et al., 2018; Haufe et al., 2019). Older adults vary not only in health status, ethnicity, and socioeconomic status, but also in health literacy and affinity to technology. These factors often interact and create a dynamic that may prevent access to AgeTech. As a result, the most complex outcomes of digital health disparities arise across social intersections, e.g., between age, gender, ethnicity, and socioeconomic status (Fang et al., 2019). Studies show that people over 65 have generally less desire and intent to use ICTs (Fang et al., 2018 b). Especially older adults with less exposure to technology throughout their work life show a lower uptake of digital health technologies (Fang et al., 2019).

Other reasons that impact technology use are health-related. Co-morbidities and functional disabilities, e.g., vision impairment or difficulties in memorising passwords, are barriers to digital access in this regard (Chen & Chan, 2013; Fang et al., 2018). This is especially the case when interfaces and systems lack the appropriate level of user-friendliness (Haufe et al., 2019).

AgeTech access and uptake is further complicated when gender is added i.e., as another layer of social complexity. In general, women use ICTs less than men, but more often for health purposes than men. Education, socioeconomic status and age are associated in this regard, since younger, more educated women are more likely to use ehealth than older, less educated men with low incomes (Fang et al., 2018). Last, individual beliefs, attitudes, and fears of technology are associated with age and generation and often play a role in preventing older adults from using AgeTech (Chen & Chan, 2013; Fang et al., 2018; Haufe et al., 2019). Older adults often consider themselves as incapable to handle the technologies due to the aforementioned factors and their perceived age or lack of digital competencies. As well, internalised-stigma including negative self-perceptions of being frail and in need of help when using AgeTech hinders technology use (Haufe et al., 2019). Such beliefs and attitudes may be the result of lack of experience with technologies, educational status, with the root of such perceptions shaped by intersectional social factors such as ethnicity, gender, and socioeconomic status.

As a consequence, the interplay of all of these technology deterrents creates a situation where individuals who can benefit the most from AgeTech are the same that people who experience the greatest access barriers (Fang et al., 2018a). A key explanation for this may be that the combined effect of social determinants is overlooked when designing AgeTech. Thus, a focus on equity already in the design process is crucial.

## Discussion and Recommendations

Although complexity management as an explicit concept has not been widely discussed so far, several strategies have been suggested for dealing with its outcomes. We will provide a short overview of these strategies, based on the main currents of the ethics of AI-based health technologies in the research literature. These strategies are often labelled as democratisation of AI-based health technologies and consist of at least three objectives (1) diversifying training data, models, and algorithms, (2) engaging relevant stakeholders as well as communities in design and implementation of AI-based health technologies, and (3) granting access to medical to all social groups and on a global scale.

### Diversifying Training Data, Models, and Algorithms (1)

Discrimination and bias in AI-based AgeTech may be a result of ignoring social determinants when selecting training data or designing models and algorithms. This issue is mainly discussed under the header of algorithmic fairness (Wawira Gichoya et al., 2021). One way to address this issue is to question existing proxies and biomarkers and to include social determinants (Walsh et al., 2020) – meaning that the socio-demographic contexts in which data has been collected must be considered. Furthermore, various social determinants should be actively integrated into the data procession process. This means that when selecting data sets for training, there needs to be assurance that the data does not focus exclusively on one social group. The epistemic scope and limits have to be evaluated based on the quality of the data in this respect (McCradden et al., 2020). If an algorithm performs poorly for specific groups, additional data from these groups should be collected and induced into the machine learning process (Walsh et al., 2020). This implies a process of model auditing throughout the design process that focusses on the reliability and validity of models as well as the assessment of confounding errors (McCradden et al., 2020). Also, upon implementation of these technologies, a local evaluation should be conducted in order to investigate hidden stratification effects (McCradden et al., 2020). A key takeaway message is that fairness should not be an afterthought or post-hoc consideration (Wawira Gichoya et al., 2021). Rather, fairness in terms of diversifying training data, models, and algorithms should be operationalised through model reporting guidelines, clinical trial guidelines, and regulatory approaches. This is an issue for AI generally and is not limited to older populations. However, the complex intersectionality of factors contributing to aging, the prevalence of ageist attitudes, and indeed increasing heterogeneity in later life create additional challenges.

### Engaging Relevant Stakeholders and Communities (2)

Participatory methods for designing AgeTech are widely seen as ways to prevent discrimination and enable equity (López Gómez & Criado, 2021). Engaging relevant stakeholders and communities may be a crucial measure throughout the life cycle of an AI-based AgeTech product. At the research stage, the perspectives of diverse end users may be required for inclusivity training data as well as user-centered technologies (Fohner et al., 2019). By using a community-engaged approach, educational aspects as well as shared decision-making are emphasised, and mutual benefit may be achieved. This approach may also foster trust in technologies, which is a crucial aspect as it pertains to the acceptability of AgeTech (Walsh et al., 2020). Using a community-engaged approach may also help to situate AI-based AgeTech in view of context-specific healthcare infrastructures and communities. Consequently, technologies tailored for the specific requirements of a given context as opposed to a one-size-fits-all-approach can mean that caregivers and care receivers are potentially more likely to use them. (Fohner et al., 2019). However, this approach has some drawbacks. Engaging stakeholders and communities can be a challenging and often very time-consuming approach, which also means more cost-intensive. Furthermore, participatory processes need a moderation in order to integrate and balance different expectations (Merkel & Kucharski, 2019). As with AgeTech itself, there is also no one-size-fits-all-approach regarding stakeholder and community engagement. In order to choose the right approach or method, it has to be clarified why a stakeholder- and community-engaging approach should be applied, what individuals or groups as well as future users will be included and at what stage of the lifecycle of the product they will be involved (Merkel & Kucharski, 2019). Another difficulty here is how to best involve older adults who are isolated, hard to reach, and who may be non-tech users in the co-development process.

### Use of Personas for User-Centered Design (2)

Developing a ‘persona’ and ‘scenario’ – a technique often used in technology design to ensure that the tech product is conducive to the end-user. A ‘persona’ is a description of a fictitious individual based on data or information from real people (Adlin & Pruitt, 2010), while scenarios provide context of the persona, which include stories of personal experience – a setting or situation in detail which a person performs a sequence of actions (possibly involving other people) to produce an outcome. In technology design, developing personas and scenarios can be viewed as a tool for considering how products can be delivered in an ethically, socially-responsible and culturally-sensitive way. The use of personas and scenarios are intended to promote our empathy with the people who we aim to serve (Jackson & Hwang, 2020). While each of us come from different backgrounds, personas can help tap into our innate human tendency to generate detailed and complex models of people and their behaviours, even when those people are fictitious (Jackson & Hwang, 2020). This is demonstrated by the ways in which we naturally try to relate to or develop understandings about fictional characters in stories or films. Using personas in teaching can help tap into this natural human aptitude that we all possess (Jackson & Hwang, 2020).

### Ethical and Responsible AgeTech (3)

Previous research has also established that factors influencing digital marginalisation are multi-faceted and that an intersectional approach needs to be adopted to fully understand how people can be disadvantaged by the increasing reliance on AgeTech (Fang et al., 2019); and in the context of AI, identify and understand if and in what ways this technology can be created with the intent to ameliorate traditional forms of marginalisation (and for whom). There are various policy pathways considered to help to ensure AgeTech access, equity and other social determinant barriers. Stix’s (2021, p. 15) notion of actionable principles for AI policy highlights how “successful actionability in policy” requires going beyond AI-based AgeTech ethics principles as a reference point, and this can be achieved, in part, by referencing transdisciplinary theoretical perspectives from disciplines of gender studies (i.e., intersectionality), urban studies (i.e., sense of place), and health sciences (i.e., digital social determinants of health). Critical analysis of existing AI design and policy by viewing the problem area through diverse theoretical viewpoints can help shed light on how we can better understand and respond to the various ways in which social positioning create distinct, and often multiple, barriers for various subgroups.

According, the three AI policy pathways presented by Stix (2021): (1) preliminary landscape assessments; (2) multi-stakeholder participation and cross-sectoral feedback; and, (3) mechanisms to support implementation and operationalisability can be supported by integrating equity-driven theoretical models and frameworks such as the Social Justice Framework for Bridging the Digital Divide, Health Equity Impact Assessment (HEIA) tool, and the Intersectional Place Perspective for AgeTech solutions (Fang et al., 2019, 2020; Ontario Ministry of Health and Long-Term Care & Toronto Central LHIN, 2009).

HEIA originated from the Health Impact Assessment methodology and has been heavily used worldwide over the past decade as a decision-making tool to facilitate the development of healthy public policy (Ontario Ministry of Health and Long-Term Care & Toronto Central LHIN, 2009). HEIA can be applied to describe the individuals and groups most affected by the progression of AI in healthcare, characterise the ways in which possible inequities can occur to support the responsible design of AI interventions and initiatives to optimise equitable access, positive ageing outcomes whilst identifying factors that may unintentionally exacerbate experiences of vulnerability and disadvantage.

The Social Justice Framework for Bridging the Digital Divide framework stems from a realist review and affirms that individuals exist within structures and systems designed by and for persons in more advantageous social positions, which creates modes of differentiation across groups and divisive access to digital resources (Fang et al., 2019). This framework may be particularly useful for recognizing and responding to the multiple layers of access and use inequities that older people might experience (Sixsmith et al., 2019), when introduced to AI interventions.

Last, the Intersectional Place Perspective designed to identify individual, social and place-based factors that shape opportunity and oppression, has been used to better understand the combined effects of the digital determinants of health and wellbeing (Fang et al., 2020, 2021). This theoretical model can help to characterise the ways in which AI can compound or alleviate inequity, through consideration of socio-cultural and environmental contextual factors that shape lifetime health and wellbeing outcomes of older people – recognising the importance of intersectionality and place. Overall, such equity-driven resources can support AI developers and shape policy pathways by providing a spotlight on how digital social determinants are operationalised in real life scenarios, which can help to ameliorate inequities associated with AI design and rollout how these might be produced and utilised across different contexts, at scale and in an ethical and sustainable way.

### Granting Access (3)

As we have seen, access barriers may arise when AI-based AgeTech does not fit the needs and resources of users. One strategy for fostering access is universal design, sometimes referred to as inclusive design or design for all (Ma et al., 2021). The aim is to reduce access barriers by designing technologies that are simple and intuitive and allow flexible use with a certain tolerance for error. This requires a well-informed design process that includes user experiences from various user groups. Another factor preventing certain user groups from access is contextual bias (Weissglass, 2021). Most AI-based AgeTech is designed in high-income countries and adapted to the structures as well as contexts of use within their respective health systems. The resulting technologies might not be fitting for the systemic or institutional conditions in low-income countries. Disclosure and validation policies might be instruments for ensuring that the context of technology design is made transparent (Weissglass, 2021). However, also in this regard, a more inclusive design process that takes diversity on a global scale into account would be needed. Another approach is to make algorithms as well as data-bases consisting of training data accessible on a global scale. This would provide researchers, clinicians, and developers from lower-income countries the opportunity to adapt technologies to their own respective contexts of use. Finally, empowering e-health literacy of older adults may reduce the digital gap, which is one of the main access barriers (Seifert et al., 2019). E-Health literacy could be improved by providing learning tools, e.g., through existing educational services. This would mean to acknowledge that older adults are not inherently technology-adverse and have both the resources and the willingness to learn, which is often ignored due to stereotypes about older adults.

## Conclusion

AI-based AgeTech has the potential to support older adults towards living a more active, independent, and healthier life. In facilitating more personalised healthcare services, AgeTech may potentially be used not only for the purpose of dealing with deficits, but rather to improve the quality of life of users. This potential, however, can only be realised in full if equity is seen as major principle for the design, implementation, and use of AI-based AgeTech. Equity is neither an add-on nor can it be an after- the- fact consideration. Rather, equity should be the guiding principle in developing AI-based AgeTech at the outset and should also guide its implementation as well as the practices it enables. This requires a well-informed design process that takes the combined effect of social determinants into account, provides accompanying measures for educating users as well as caregivers, and defines regulatory approaches that address the issues of bias and discrimination.
