# Correction: Equity in AgeTech for Ageing Well in Technology-Driven Places: The Role of Social Determinants in Designing AI-based Assistive Technologies

**DOI:** 10.1007/s11948-022-00424-y

**Published:** 2022-12-13

**Authors:** Giovanni Rubeis, Mei Lan Fang, Andrew Sixsmith

**Affiliations:** 1 Division Biomedical and Public Health Ethics, Department General Health Studies, Karl Landsteiner University of Health Sciences, Dr.-Karl-Dorrek-Straße 30, 3500 Krems, Austria; 2 School of Health Sciences, University of Dundee Nethergate, DD1 4HN Dundee, Scotland, UK; 3 Department of Gerontology, Simon Fraser University Vancouver Harbour Centre, Room, 2800, Vancouver, BC V6B 5K3 Canada

**Correction to: Science and Engineering Ethics (2022) 28:49**

10.1007/s11948-022-00397-y

In this article the author name Mei Lan Fang was incorrectly written as Mei Lang Fang.

The original article has been corrected.

